# Patient-reported quality of life and echocardiographic recovery after coronary angioplasty: A prospective questionnaire-based study

**DOI:** 10.6026/973206300221919

**Published:** 2026-04-30

**Authors:** Sanathraj Patlu Devaraj, Moha Merazul Ashekin, Bhawana Birud, Amina Misbah, Saravenensandeep V Pathmanathan

**Affiliations:** 1 Department of Medicine, Bangalore hospital, Kengeri, Karnataka, India; 2 Department of Internal Medicine, Dhaka, Bangladesh; 3 Department of Physiology, NKP Salve Institute of Medical sciences & RC and LMH, Nagpur, India; 4 Department of Emergency Medicine and Internal Medicine, DHA, Aster Medcity, Dubai, United Arab Emirates; 5 Department of Orthopedics, Mater Dei Hospital, Msida, Malta

**Keywords:** Quality of life (QoL), angioplasty, echocardiography, patient-reported outcomes (PRO), survey

## Abstract

Despite widespread use of percutaneous coronary intervention (PCI), post-procedural follow-up primarily emphasizes imaging outcomes while patient-reported quality of life (QoL) remains under-integrated into recovery assessment. Therefore, it is of interest to evaluate 120 patients undergoing elective angioplasty and examined the association between longitudinal QoL scores and echocardiographic recovery at 6 and 12 months. Mean QoL improved from 62.3 ± 14.5 at baseline to 78.5 ± 12.3 at 12 months (p < 0.001), alongside significant improvements in left ventricular ejection fraction, global longitudinal strain and wall motion score index (p < 0.001). Improvement in QoL demonstrated a moderate positive correlation with change in LVEF (r = 0.46) and an inverse correlation with WMSI improvement (r = -0.38). Thus, we show that subjective recovery parallels objective ventricular improvement and support integration of structured QoL assessment into routine post-angioplasty care.

## Background:

Coronary artery disease (CAD) is treated and managed through a procedure called percutaneous coronary intervention (PCI), which has been demonstrated to improve not only the overall survival of CAD patients but also the severity of symptoms caused by their disease [1]. As advances have been made with PCI we have continued to see a number of patients with residual functional limitations, social issues and decreased health-related quality of life (HRQoL) after undergoing a PCI procedure [2]. PCI outcomes reported by interventional cardiologists tend to be predominantly focused around traditional imaging-related metrics such as LVEF (left ventricular ejection fraction), strain values and successful angiographic results [3]. In contrast, patient-reported outcomes (PRO) are not consistently included in routine follow-up visits of PCI patients [4]. Evidence presented in recent studies has demonstrated that the HRQoL of PCI patients at the time of their hospital discharge has an independent association with rehospitalisation, cardiovascular events and overall long-term health status [5]. Whereas improvements in ventricular function have accompanied consistent symptomatic improvements, there is still an insufficient understanding of how the subjective well-being of patients relates to the objective echocardiographic measures of recovery from a PCI procedure [6]. Restoration of myocardial function can be quantified through echocardiographic measurements (such as LVEF, GLS, WMSI), but the current echocardiography assessments do not fully define a patient's return to normal physical function, emotional well-being, or self-perceived health [7]. Therefore, incorporating structured measures of patient-reported QoL into the process of patient follow-up would allow interventionists to gain a better understanding of recovery patterns after a PCI procedure [8]. Therefore, it is of interest to evaluate the HRQoL of patients after PCI and how it relates to echocardiographic findings over time (6 months and 1 year post- PCI).

## Materials and Methods:

The authors of this article describe a prospective observational study of 120 adult patients aged 30-80 years who underwent a successful elective percutaneous coronary intervention at a tertiary cardiac centre in Australia between January and June 2023. This study was conducted using the premise that coronary artery disease (CAD) is often associated with decreased quality of life (QoL) in patients who have it and that there are valid ways to assess QoL. Patients with significant residual coronary stenosis (>50% in non-treated vessels), advanced heart failure (NYHA III-IV), acute myocardial infarction (<30 days), or any major non-cardiac comorbidities were excluded from the study. Ethical approval for the study was received from the Institutional Ethics Committee and all patients provided written informed consent for participation in the study. All patients were assessed prior to discharge from the hospital using a validated 20-item health- related QoL questionnaire that evaluated physical function, emotional wellbeing, and symptom burden and activity limitation. Responses to the questionnaire were converted into scores on a scale of 0 (worst QoL) to 100 (best QoL), with higher scores indicating a better QoL. Follow-up assessments were performed at six and 12 months, during which patients repeated the QoL questionnaire and underwent transthoracic echocardiography to assess echocardiographic measures: left ventricular ejection fraction (LVEF), global longitudinal strain (GLS) and wall motion score index (WMSI). The changes in QoL and echocardiographic measures from the baseline to 12 months were calculated and expressed as descriptive statistics: mean±SD or frequency. The relationship between the change in QoL and change in echocardiographic measures was assessed using Pearson correlation analysis. Identifying potential independent predictors of QoL improvement at 12 months was performed using multivariable linear regression, adjusting for age, sex, baseline LVEF and completeness of revascularization. Results were considered statistically significant at p<0.05 and data analyses were performed using SPSS version 26.0.

## Results:

A total of 120 patients were included in the analysis, of whom 60% were male and the mean age was 62.4 ± 8.9 years. Hypertension was present in 71.7%, diabetes mellitus in 53.3% and dyslipidemia in 58.3% of participants. The mean baseline left ventricular ejection fraction (LVEF) was 48.1 ± 7.2%. The mean baseline QoL score was 62.3 ± 14.5. At 6 months, QoL increased to 72.1 ± 13.2 and at 12 months it further increased to 78.5 ± 12.3 (p < 0.001). Echocardiographic parameters showed significant improvement at 12 months, with LVEF increasing to 54.6 ± 6.8%, GLS improving by 2.4% and WMSI decreasing from 1.42 ± 0.23 to 1.28 ± 0.18 (p < 0.001). Patients with greater LVEF and GLS improvement demonstrated higher QoL gains. Correlation analysis revealed moderate associations between QoL improvement and changes in ventricular function. Multivariable regression identified younger age, higher baseline LVEF and completeness of revascularisation as significant predictors of QoL improvement. Table 1 (see PDF) shows that the mean age was 62.4 ± 8.9 years, 60% were male, 71.7% had hypertension, 53.3% had diabetes mellitus and baseline LVEF was 48.1 ± 7.2%, indicating moderately impaired ventricular function at presentation. Table 2 (see PDF) indicates that mean QoL increased from 62.3 ± 14.5 at baseline to 72.1 ± 13.2 at 6 months and 78.5 ± 12.3 at 12 months, representing a 26.0% improvement from baseline (p < 0.001). Table 3 (see PDF) demonstrates that LVEF improved by 6.5 ± 3.1%, GLS improved by 2.4 ± 1.2% and WMSI decreased by 0.14 ± 0.09 at 12 months, all with statistical significance (p < 0.001). Table 4 (see PDF) shows that patients with LVEF improvement ≥5% had greater QoL gain (19.4 ± 8.2) compared to those with <5% improvement (9.7 ± 7.9) and similar higher QoL gains were observed in patients with GLS improvement ≥2% and WMSI improvement ≥0.1 (p < 0.001). Table 5 (see PDF) depicts a moderate positive correlation between QoL improvement and ΔLVEF (r = 0.46), a mild positive correlation with ΔGLS (r = 0.31) and a moderate inverse correlation with ΔWMSI (r = -0.38), all statistically significant. Table 6 (see PDF) highlights that younger age (β = -0.12, p = 0.003), higher baseline LVEF (β = 0.16, p = 0.004) and completeness of revascularisation (β = 0.24, p < 0.001) independently predicted greater QoL improvement. Table 7 (see PDF) demonstrates that symptom relief (70%), return to daily activities (55%) and psychological confidence (48%) were commonly associated with improved QoL, whereas medication concerns (23%) and persistent fatigue (15%) were associated with reduced QoL perception.

## Discussion:

The findings from this prospective study show that elective coronary angioplasty results in significant and sustained increases in both echocardiographic parameters and patient-reported quality of life (QoL). Over the 12 months of the study, there was an increase of 26% in mean QoL for the patients, in addition to a significant improvement in echocardiographic parameters such as left ventricular ejection fraction (LVEF), global longitudinal strain (GLS) and wall motion score index (WMSI) [9]. These results demonstrate a moderate correlation between the changes in QoL and the recovery of the left ventricle after an angioplasty, indicating that people's happiness can reflect recovery of the left ventricle [10]. The improvement in LVEF and myocardial strain has consistently been associated with relief of symptoms and improvement in functioning for patients who have undergone coronary revascularisation. This study adds to the existing literature by quantifying how QoL changes over time are associated with echocardiographic recovery in the same cohort of patients [11]. Patients who achieved an improvement of 5% or greater in LVEF had nearly twice the amount of improvement in QoL than patients who did not, suggesting that there may be a clinically relevant threshold effect. Furthermore, the correlation between changes in GLS and WMSI with respect to QoL scores shows that improving myocardial recovery is functionally significant [12]. Importantly, improvements in QoL do not solely depend on changes in imaging measurements. Qualitative findings indicate that relief of symptoms; resuming daily activities and having psychological confidence are all important components of perceived recovery [13]. These areas are indicative of regaining autonomy and emotional stability, which cannot be completely captured by ventricular parameters. Current literature highlights that health-related QoL after percutaneous coronary intervention (PCI) is an independent predictor of rehospitalisation and long-term cardiovascular outcomes, which points to the prognostic value of QoL beyond that of traditional imaging endpoints [14]. Additionally, multiple regression analyses identified younger age, higher baseline LVEF and having had a complete revascularisation as being independent predictors of QoL gain [15]. The strongest predictor is complete revascularisation, which implies that anatomic success provides physiological and experiential benefits. Similar to the present study, other studies have also shown that patients who undergo complete versus incomplete revascularisation experience improved functional and QoL outcomes. These predictors of QoL gain can potentially help to identify patients who may benefit from closer monitoring or additional rehabilitation strategies [16]. The present study adds to the current body of knowledge by integrating an objective assessment of QoL over time with a structured approach to the longitudinal assessment of echocardiographic outcomes. While echocardiography is typically performed following PCI, patient-reported outcomes (PRO) are not consistently collected or integrated into longitudinal care. This correlation between subjective measures of recovery and actual recovery of the left ventricle provides objective, empirical justification for the incorporation of QoL assessment into routine post-angioplasty care pathways. Taking this approach to rehabilitative care would allow for a more thorough evaluation of the effectiveness of interventions and therefore, improve patient-centred outcomes. Several limitations to this study should be acknowledged. The study was conducted at a single institution and had a moderate sample size, which limits the broad applicability of these results. Further, all QoL measurements were based on self-reports and were potentially affected by psychosocial factors. Lastly, the echocardiographic evaluations in this study were based on visual images of the patient's left ventricular walls and did not capture the entire microvascular perfusion/perfusion burden that could exist. As a result, future multicentre studies including more advanced imaging and rehabilitation techniques are warranted. In summary, these results support the combined assessment of both patient-reported outcomes and imaging-based assessments following coronary angioplasty. The integration of these two methods will allow for more individually tailored follow-up strategies and potentially greater improvements in long-term recovery.

## Conclusion:

Improvement in patient-reported quality of life after coronary angioplasty closely parallels objective recovery in ventricular function. Moderate correlations between QoL gain and echocardiographic improvement support the integration of structured patient-reported outcome measures into routine post-PCI follow-up. Thus, an imaging-based assessment provides a more comprehensive evaluation of recovery and may enhance patient-centred cardiac care.
